# The Characterization and Cytotoxic Evaluation of *Chondrosia reniformis* Collagen Isolated from Different Body Parts (Ectosome and Choanosome) Envisaging the Development of Biomaterials

**DOI:** 10.3390/md22020055

**Published:** 2024-01-24

**Authors:** Miguel S. Rocha, Catarina F. Marques, Ana C. Carvalho, Eva Martins, Alexander Ereskovsky, Rui L. Reis, Tiago H. Silva

**Affiliations:** 1 3B’s Research Group, I3Bs—Research Institute on Biomaterials, Biodegradables and Biomimetics, University of Minho, Headquarters of the European Institute of Excellence on Tissue Engineering and Regenerative Medicine, AvePark, Parque de Ciência e Tecnologia, Zona Industrial da Gandra, Barco, 4805-017 Guimaraes, Portugal; miguel.rocha@i3bs.uminho.pt (M.S.R.); catarina.marques@i3bs.uminho.pt (C.F.M.); anacpcarvalho@gmail.com (A.C.C.); eva.biotec@gmail.com (E.M.); rgreis@i3bs.uminho.pt (R.L.R.); 2 ICVS/3B’s–PT Government Associate Laboratory, 4806-909 Braga/Guimaraes, Portugal; 3 Institut Méditerranéen de Biodiversité et d’Ecologie Marine et Continentale (IMBE), Aix Marseille University, Avignon University, Centre National de la Recherche Scientifique (CNRS), Institut de Recherche pour le Développement (IRD), 13007 Marseille, France; alexander.ereskovsky@imbe.fr; 4 Faculty of Biology, Department of Embryology, Saint Petersburg State University, 199034 Saint Petersburg, Russia; 5 N.K. Koltzov Institute of Developmental Biology of Russian Academy of Sciences, 119334 Moscow, Russia

**Keywords:** collagen, *C. reniformis*, marine sponges, ectosome, choanosome

## Abstract

*Chondrosia reniformis* is a collagen-rich marine sponge that is considered a sustainable and viable option for producing an alternative to mammalian-origin collagens. However, there is a lack of knowledge regarding the properties of collagen isolated from different sponge parts, namely the outer region, or cortex, (ectosome) and the inner region (choanosome), and how it affects the development of biomaterials. In this study, a brief histological analysis focusing on *C. reniformis* collagen spatial distribution and a comprehensive comparative analysis between collagen isolated from ectosome and choanosome are presented. The isolated collagen characterization was based on isolation yield, Fourier-transformed infrared spectroscopy (FTIR), circular dichroism (CD), SDS-PAGE, dot blot, and amino acid composition, as well as their cytocompatibility envisaging the development of future biomedical applications. An isolation yield of approximately 20% was similar for both sponge parts, as well as the FTIR, CD, and SDS-PAGE profiles, which demonstrated that both isolated collagens presented a high purity degree and preserved their triple helix and fibrillar conformation. Ectosome collagen had a higher OHpro content and possessed collagen type I and IV, while the choanosome was predominately constituted by collagen type IV. In vitro cytotoxicity assays using the L929 fibroblast cell line displayed a significant cytotoxic effect of choanosome collagen at 2 mg/mL, while ectosome collagen enhanced cell metabolism and proliferation, thus indicating the latter as being more suitable for the development of biomaterials. This research represents a unique comparative study of *C. reniformis* body parts, serving as a support for further establishing this marine sponge as a promising alternative collagen source for the future development of biomedical applications.

## 1. Introduction

Collagen is widely known as the major structural protein of the human body and is present in various connective tissues, representing one-third of the total protein content [1]. Due to its particular characteristics, such as the presence of several proteoglycan binding domains, growth factors, and other cell signaling molecules, collagen is considered a great material for Tissue Engineering and Regenerative Medicine (TERM) applications [2,3,4,5]. Although the main industrial collagen sources for biomedical applications are still of mammalian origin, in recent decades, marine-origin collagens have been receiving growing attention and are now considered high-value materials. This alternative and promising source has several advantages when compared with its mammalian counterparts, namely the prevention of zoonosis transmission such as BSE (bovine spongiform encephalopathy) and FMD (Foot and mouth disease), as well as of immunogenic reactions [6,7]. Additionally, religious and ethical constraints regarding porcine or bovine derivatives, namely Hindu, Muslim, and Jewish cultures, are avoided while having a lower production cost and higher yields than recombinant collagen [8,9]. In fact, the high demand for marine collagen encouraged the search for environmentally friendly sources, either derived from sustainable origins or from fish by-product valorization [10,11,12]. Recently, many TERM approaches have already been successfully developed using marine-origin collagenous materials, ranging from wound healing to drug delivery, as well as cosmetics [10,11,13,14,15,16]. 

*Chondrosia reniformis* is a demosponge commonly found in the shallow waters of the Mediterranean Sea and the South-West cost of the Atlantic Ocean; it lacks endogenous spicules and is particularly rich in collagen [17]. Its body is constituted by two main distinct zones: the ectosome and the choanosome. The ectosome, or cortex, is the cortical zone of the sponge composed of a layer of exopinacocytes surrounding the densely interwoven bundles of fibrils of collagen; it is poorly irrigated by the incurrent aquiferous system canals [18]. The choanosome is the internal zone of the sponge, which contains the choanocyte chambers lined by choanocytes and is filled with a densely interlaced network of inner vessels surrounded by sheaths of cortical tissue that form a dense three-dimensional stroma [18]. 

Recently, an integrated mariculture method using *C. reniformis* was developed, enabling a sustainable and high-yielding marine collagen production process that is adaptable to seawater environments combined with organic matter sources such as fish culture or sewage outfall [12]. This progress makes this collagen-rich marine sponge one of the most promising collagen sources of the present time, as its mariculture has a positive effect on the surrounding marine environment while allowing for an environmentally friendly animal collection. Due to both its high collagen content and its ability to reversely modulate the mechanical properties of its mesohyl, research on this highly collagenous animal has been encouraged [19,20,21,22,23]. It has been described that *C. reniformis* possesses collagens similar to type I and type IV and that their distribution in ectosome and choanosome is distinct [24,25]. Type IV collagen was reported to be expressed mainly in the ectosome, while collagen that was isolated from the whole *C. reniformis* body presented similarities to bovine type I collagen, namely in the amino acid composition and infrared spectra [24,25]. Nevertheless, *C. reniformis* collagen isolation procedures, performed employing neutral buffer solutions or disaggregating solutions and using the sponge whole body or just the ectosome, always described the isolated intact fibrils as resembling collagen type I [19,24,26,27]. Other studies used a green extraction process based on water acidified with CO_2_ for the high-yielding isolation of collagen/gelatin from the sponge’s whole body, which presented similar properties to collagen isolated from other marine sources [20,28]. Nonetheless, despite these promising results, *C. reniformis* collagens have not been thoroughly characterized and are not commonly used for TERM applications, although some works have been developed [22,29,30]. In fact, previous studies focusing on this issue have employed collagen isolated from the whole cortex or from specific body parts (ectosome or choanosome), but no comparative study has ever been performed. The only study presented so far lacks a thorough assessment of the physicochemical properties of the isolated collagens and presents no data regarding possible cytotoxic effects [23].

In this work, we examined two distinct *C. reniformis* body parts—the ectosome and choanosome—via histology, focusing on their collagen content and spatial organization. Furthermore, collagen isolated from both body parts was studied in more detail to assess its physicochemical properties as well as its biological performance. Following an established collagen isolation procedure, cytotoxicity assays using L929 cells employing different concentrations of collagen isolated separately from the ectosome and choanosome were performed for the first time, thus evaluating their suitability for the development of biomedical applications, particularly in tissue engineering strategies. These data are important as they can add value to collagen isolated from this sponge, which is considered a sustainable collagen source [12]. This study provides valuable information for the establishment of *C. reniformis* as an ecological and biomedically relevant source of collagen and is paramount for the future development of marine collagen-based biomaterials.

## 2. Results

### 2.1. Histological Characterization of C. reniformis

**Figure 1:**
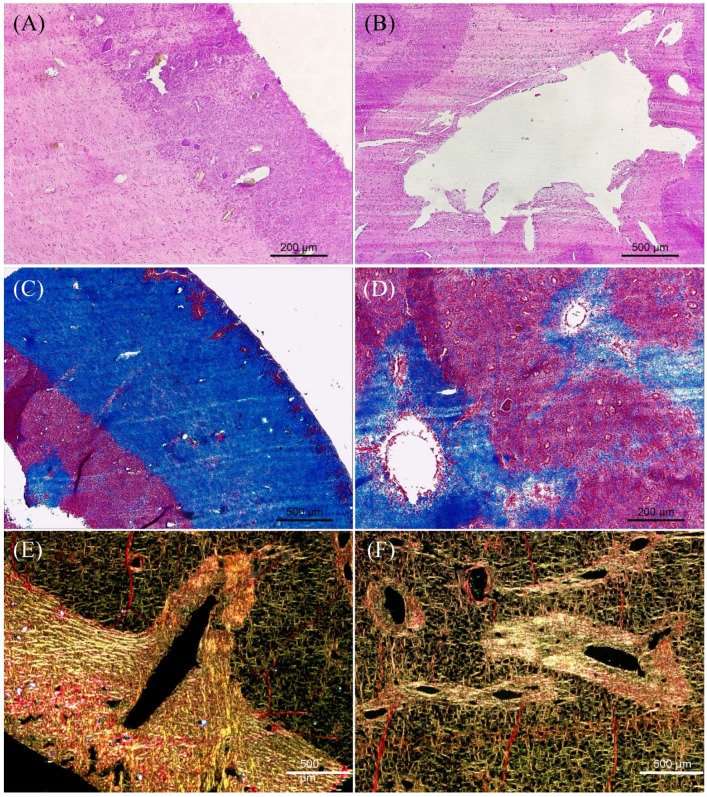
Histological cross-transversal sections of *C. reniformis* mesohyl on paraffin sections. (**A**) Section stained with H&E showing transition between ectosome (ec) on the right and choanosome (cho) on the left. (**B**) Section stained with H&E showing a big canal (ca) of aquiferous system present in the choanosome. (**C**) Section stained with Masson’s trichome showing transition between ectosome (ec) on the right and choanosome (cho) on the left. (**D**) Section stained with Masson’s trichome showing choanosome and some canals (ca) of aquiferous system. (**E**) Section stained with Picrosirius red showing transition between ectosome (ec), on the left, and choanosome (cho), on the right, and the presence of a canal (ca) of aquiferous system. (**F**) Section stained with Picrosirius red showing canals (ca) of aquiferous system present in the choanosome.

Histology was performed to analyze the overall anatomy and microarchitecture of the *Chondrosia reniformis* mesohyl (Figure 1). Images of the transversal sections comprising the transition between ectosome and choanosome revealed differences regarding collagen content and organization (Figure 1). Hematoxylin and eosin (H&E) staining provided a general view of the sponge anatomy, allowing us to analyze the ectosome and choanosome structure and morphology (Figure 1A,B). It revealed a clear and easily distinguishable difference in the density of the extracellular matrix (ECM) between both body parts, as the ECM of the ectosome was denser than the choanosome (Figure 1A). Additionally, the areas surrounding the aquiferous system canals present in the choanosome had a higher ECM density than the rest of the tissue (Figure 1B). Moreover, some rock or sand debris was found incorporated exclusively in the outer zone of the sponge, a phenomenon that was previously reported (Figure 1A) [31]. 

Collagen-specific Masson’s Trichrome staining showed that the ectosome was mostly constituted by collagen (stained blue), contrasting with choanosome, which had a much lower collagen content (Figure 1C). The highly collagenous extracellular framework present in the ectosome supports the sponge body and is similar to other metazoan taxa collagenous connective tissues [18]. In the choanosome, the collagen present that surrounds the canals provides stability to the sponge aquiferous system (Figure 1D). Picrosirius red staining, which allows for the visualization of the collagen fibrils’ orientation, abundance, and thickness, showed that *C. reniformis*’s whole body consisted of densely interwoven bundles of collagen fibrils, although much more concentrated in the ectosome and around the aquiferous system canals (Figure 1E,F). Collagen fibers present in the ectosome and around the canals of the aquiferous system were bright yellow as they were larger and thicker, while collagen fibers present in the choanosome were thinner as they displayed a greenish color [32]. This observation agreed with Masson’s Trichrome results, as it demonstrated that the ectosome and the areas surrounding the aquiferous system canals were the richest regions in collagen and presented thicker fibers. This may hint at different collagen types being present in the distinct regions. Despite being known to possess a mainly collagenous body, evidenced by the collagen fibrils being present throughout the entire *C. reniformis* mesohyl, it was clear that there were differences in collagen content depending on the body part. This might have an impact on collagen isolation procedures, as yields and isolated collagen purity may be affected.

### 2.2. Collagen Characterization

Collagen has been widely used for biomedical applications; hence, it is imperative to isolate a high-purity and high-quality material with stable properties that match the requirements of the specific application intended before it can be validated and employed. Therefore, collagen isolated separately from the *C. reniformis* ectosome and choanosome was thoroughly characterized regarding its physicochemical properties.

**Figure 2:**
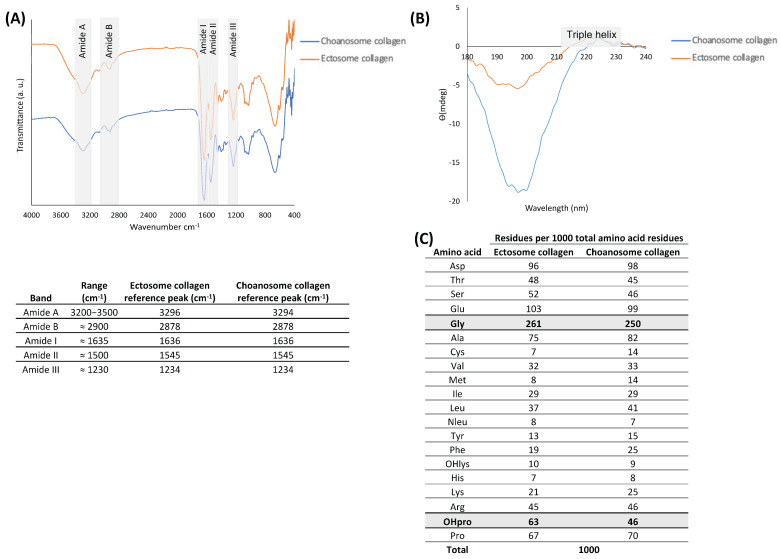
(**A**) Fourier transform infrared (FTIR) spectra and reference peaks and peak assignments of ectosome (orange line) and choanosome (blue line) collagens. (**B**) Circular dichroism (CD) spectra of ectosome (orange line) and choanosome (blue line) collagens. (**C**) Amino acid content of ectosome and choanosome collagens.

Fourier transform infrared (FTIR) spectra of collagen from *C. reniformis* ectosome and choanosome were very similar to each other, suggesting that their chemical composition was identical (spectra and table in Figure 2A). Both FTIR spectra presented the peaks representing amide A, amide B, amide I, amide II, and amide III bonds, which are commonly associated with collagen and indicative of the secondary structure of different materials [20,26,28]. The amide A broadband is associated with N–H stretching, demonstrating the presence of intermolecular hydrogen bonds, while the amide B band represents the CH_3_ asymmetrical stretch. The amide I peak is associated with the proteins’ carbonyl group stretching vibrations (C=O), the amide II peak results from the N–H bending vibration coupled with the C–N stretching vibration, and the amide III band is related to C–H stretching. The band representing amide I is the most intense and sensitive, and it can be considered a useful marker for the analysis of the protein secondary structure, while the amide III band is considered a collagen fingerprint as it is credited to the characteristic collagen repeating tripeptide Gly-X-Y [33]. Taking this into account, in both spectra, the reference peaks of collagen were clearly visible.

Circular dichroism (CD) spectra of *C. reniformis* collagen from the ectosome and choanosome in the wavelength of 180 to 240 nm are shown in Figure 2B. This technique enabled us to evaluate the secondary structure of the isolated collagens and confirm if the isolation process did not denature the proteins. Both collagens spectra presented a negative peak around 200 nm and a positive peak around 225 nm, which is the characteristic profile of the collagen triple helix conformation [34,35,36]. Preservation of the characteristic triple helix conformation is important since its loss by denaturation has an undesirable detrimental effect on the performance of biological molecules [37,38]. 

Figure 2C shows the results of the amino acid measurement of ectosome and choanosome collagens as the molar ratio of a given amino acid with regard to 1000 total amino acid residues, which is the approximate value of amino acids in each collagen alpha chain [8]. In both samples, the most abundant amino acid was glycine (Gly), accounting for roughly one-quarter, in line with the findings obtained previously [20,27]. Glutamic acid (Glu) and aspartic acid (Asp) were the second and third most abundant amino acids, respectively, in both samples. Additionally, OHpro, which results from the hydroxylation of proline and is a characteristic post-translation modification of collagen proteins used as a marker of collagen presence in protein extracts, was present more abundantly in ectosome collagen than in choanosome [39]. OHpro and Pro (pyrrolidine acids) are known to enhance the thermal stability of the triple helix conformation conferred by inter-chain hydrogen bonding between the carbonyl groups of the polypeptides, with marine-origin collagens typically exhibiting a lower denaturation temperature than terrestrial mammals collagens [40]. Moreover, the hydroxylation degree is a significant parameter to evaluate the collagen thermal stability and helix structure [41]. The hydroxylation degree of ectosome collagen (48.5%) was higher than choanosome collagen (39.7%), hinting at the higher thermal stability of this collagen molecule [42]. Thermal stability of the collagen triple helix conformation is paramount, as this protein is an essential structural compound that has the ability to support various connective tissues [8].

**Figure 3:**
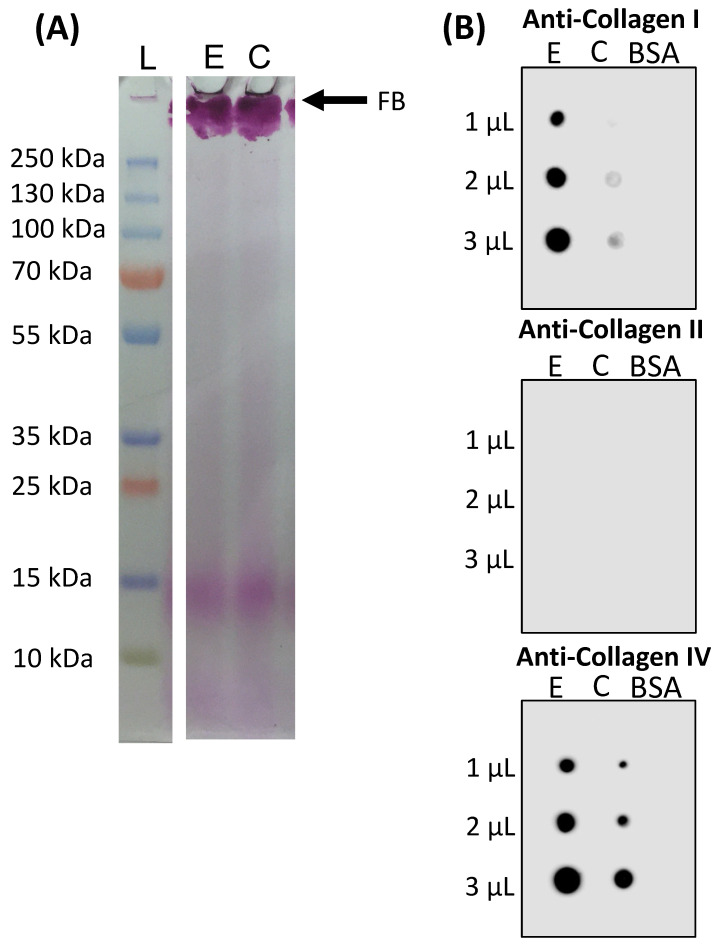
(**A**) Sodium dodecyl sulfate-polyacrylamide gel electrophoresis (SDS-PAGE) pattern of ectosome and choanosome collagens: L: protein marker; E: ectosome collagen; C: choanosome collagen; FB: fibrillar collagen. (**B**) Dot blot assay verifying the presence of collagen type I, II, and IV in the ectosome (E) and choanosome (C) collagens; bovine serum albumin (BSA) was used as negative control.

Both collagens were screened on an electrophoresis gel, which allowed the separation of protein chains by their molecular weight (Figure 3A). Observing the obtained SDS-PAGE profiles, it was possible to detect both samples on the high molecular region (stacking gel), as they were not able to penetrate the separating gel. This was due to the collagen’s high molecular weight, around 300 kDa, as the collagen isolation process employed in this work preserved collagen in its fibrillar form [8,23,43]. Additionally, although Coomassie blue staining did not allow for the visualization of any bands (results not shown), the glycoprotein stain employed clearly dyed both samples, indicating that they were highly glycosylated. The low-molecular-weight bands present around 20 kDa were possibly collagen peptides resulting from hydrolysis or a low-concentration contaminant protein co-isolated with collagen. However, it is probable that these bands corresponded to collagen peptides since they were dyed as well demonstrating their highly glycosylated nature.

The isolated collagens were analyzed for the collagen types present in their constitution by dot blot, and the results are depicted in Figure 3B. Although *C. reniformis* has been described as possessing collagen types I and IV, antibodies specific to collagen types I, II, and IV were employed in this assay. The results confirmed the presence of collagen type I and IV in both body parts, while collagen type II was not detected, which is in accordance with previous reports [24,25]. Collagen type IV was present in high quantities in both samples, although it appeared to be present in a slightly higher quantity in the ectosome since the detection was moderately stronger than in the choanosome. Regarding collagen type I, ectosome had an evidently higher quantity when compared to choanosome, as it presented a heavy detection similar to collagen type IV, indicating that this body part was constituted by equal amounts of both collagen types. However, the amount of collagen type I detected in choanosome collagen appeared to be residual as the signal was barely detectable, indicating that it was mainly constituted by type IV collagen.

The collagen isolation yield of each sponge body part is evaluated and represented in Table 1. It was observed a recovery of around 20% of the total wet weight of the sponge tissue for collagen isolation procedures from both body parts, with no significant differences observed. Accordingly, on average, 0.2 g of collagen extract can be obtained from 1 g of dried *C. reniformis* employing this collagen isolation protocol. Although histology results point to the fact that ectosome is richer in collagen than choanosome, it had no effect on the final collagen isolation yield. 

### 2.3. Collagen Biological Assessment

**Figure 4:**
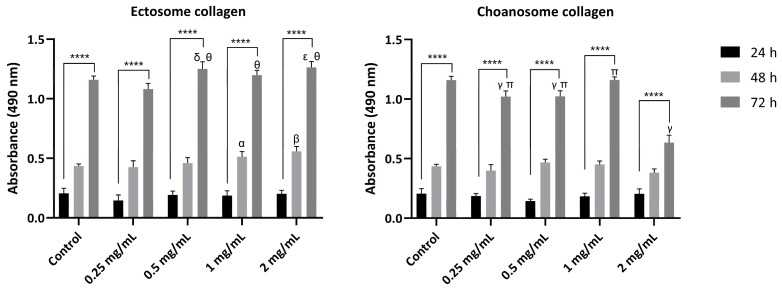
Metabolic activity of untreated L929 cells (control) and treated with ectosome and choanosome collagen dissolved at different concentrations (0.25 mg/mL, 0.5 mg/mL, 1 mg/mL, and 2 mg/mL) for 24 h, 48 h, and 72 h as determined by MTS assay. Data are mean ± standard deviation (*n* = 3, statistical significance for * *p* ≤ 0.05; ** *p* ≤ 0.01 and **** *p* ≤ 0.0001, and symbols denote statistical differences: α (*) and β (****) compared with 48 h of control, δ (*), ε (**), and γ (****) compared with 72 h of control, θ (****) compared with 72 h of 0.25 mg/mL ectosome collagen, and π (****) compared with 72 h of 2 mg/mL choanosome collagen).

**Figure 5:**
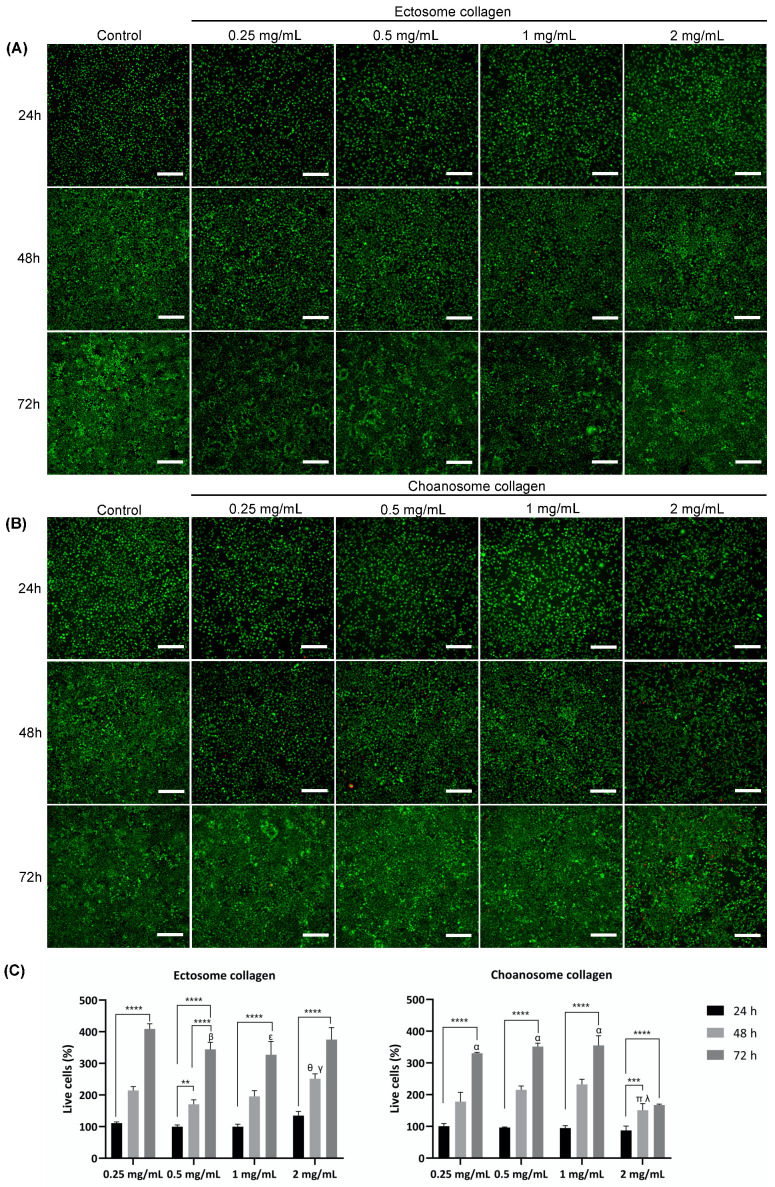
(**A**) Microscopy of live/dead assay of untreated L929 cells (control) and L929 cells treated with ectosome collagen dissolved at different concentrations (0.25 mg/mL, 0.5 mg/mL, 1 mg/mL, and 2 mg/mL) for 24 h, 48 h, and 72 h. Viable cells were stained with calcein-AM (green), and dead cells with PI (red). Scale bar: 200 μm. (**B**) Microscopy of live/dead assay of untreated L929 cells (control) and L929 cells treated with choanosome collagen dissolved at different concentrations (0.25 mg/mL, 0.5 mg/mL, 1 mg/mL, and 2 mg/mL) for 24 h, 48 h, and 72 h. Viable cells were stained with calcein-AM (green) and dead cells with PI (red). Scale bar: 200 μm. (**C**) Quantitative analysis of fluorescence of viable cells (%) treated with ectosome and choanosome collagen at different concentrations (0.25 mg/mL, 0.5 mg/mL, 1 mg/mL, and 2 mg/mL) at 24 h, 48 h, and 72 h. Results are expressed as percentages relative to the control (viable cells of control at 24 h). Data are mean ± standard error (*n* = 6, statistical significance for * *p* ≤ 0.05; ** *p* ≤ 0.01; *** *p* ≤ 0.001 and **** *p* ≤ 0.0001, and symbols denote statistical differences: β (*) and ε (**) compared with 72 h of 0.25 mg/mL ectosome collagen, θ (***) and γ (*) compared with 48 h of 0.5 mg/mL and 1 mg/mL ectosome collagen, respectively, α (****) compared with 72 h of 2 mg/mL choanosome collagen and π (***) and λ (****) compared with 48 h of 0.5 mg/mL and 1 mg/mL choanosome collagen, respectively).

After a thorough characterization of the physicochemical properties of the isolated collagens, a biological assessment was performed to understand if the previously detected differences between the collagens influence their biological performance. The cytotoxicity of both isolated collagens was evaluated by assessing the in vitro biological performance of fibroblast-like cells in contact with different collagen concentrations. The metabolic activity and viability of cells cultured in the presence of collagen dissolved in culture medium at different concentrations (0.25, 0.5, 1, and 2 mg/mL) were assessed via MTS and live/dead assays, respectively, and compared with cells cultured in culture medium without added collagen (Figure 4 and Figure 5).

Cell metabolic activity was assessed up to 72 h in the presence of different collagen concentrations and in their absence, the latter being considered the negative control (Figure 4). The results demonstrated that collagen isolated from ectosome did not detrimentally affect the metabolic activity of the cells, as in the presence of this collagen, the cell metabolic activity increased in a concentration- and time-dependent manner, determining that there was no cytotoxic effect. In fact, higher collagen concentrations (0.5, 1, and 2 mg/mL) induced a significant increase in cell metabolism relative to the control at 48 and 72 h. 

Concerning collagen isolated from choanosome, the obtained results contrasted with the obtained with ectosome collagen. Even at the lowest concentration (0.25 mg/mL), there was an inhibitory effect on cell metabolism at 72 h relative to the control. At the highest concentration (2 mg/mL), the detrimental effect on cell metabolism at 72 h was even more evident. Although at 24 h and 48 h, no effect was detected, at the last time point, cell metabolism significantly decreased when compared with the control and with the other tested choanosome collagen concentrations tested. This result clearly highlighted the choanosome’s collagen-negative effect on cell metabolism, especially at 2 mg/mL.

To further complement these results, cell viability was determined via live/dead assay (Figure 5). It was possible to observe that the green signal, related to living cells, greatly increased over time in all ectosome collagen concentrations tested, demonstrating that the cells were alive and proliferated in accordance with the MTS results (Figure 5A). There was a residual red signal detected as well, related to dead cells, but the great majority of cells were alive. At 72 h, it was observed that viable cells had essentially proliferated throughout the whole culture plate. Regarding the choanosome collagen up to a 1 mg/mL concentration, the results were almost comparable to the control (Figure 5B). The green signal increased over time while the red signal was minimal, indicating that cells were alive and proliferated, although at a slightly slower rate than in the control and ectosome collagen. However, at the highest choanosome collagen concentration tested (2 mg/mL), the green signal increase was reduced while the red signal slightly increased over time. In fact, at 72 h, it was possible to see an increment of dead cells and considerable areas with no cells. Since at each time point, it was necessary to remove the culture medium before adding the culture medium with calcein-AM and PI, it is plausible that the areas with no cells were previously occupied with dead cells, which were removed during the wash. This result clearly demonstrated that cell viability was negatively affected when in contact with choanosome collagen at 2 mg/mL, which is in accordance with MTS results. Additionally, a quantitative analysis of the live/dead results was performed using the live cells at 24 h of control as reference (Figure 5C). Although this evaluation is known to be less precise than the MTS assay due to counting errors that may be involved, the obtained results were generally in agreement with the MTS data. On one hand, the highest concentration tested of ectosome collagen presented the highest percentage of viable cells. On the other hand, the highest concentration tested of choanosome collagen had the lowest number of live cells, a detrimental effect detected at 48 h and 72 h. The inhibitory effect of this collagen at 2 mg/mL was obvious as the number of live cells was significantly lower at 48 h and barely increased from 48 h to 72 h. This reinforces the hypothesis that choanosome collagen was harmful to the cells tested.

## 3. Discussion

Although in recent years, some biomedical, cosmetical, and drug delivery applications have been developed using collagen from *Chondrosia reniformis*, an in-depth study of collagen isolated from distinct sponge body parts is still lacking [21,22,27,29]. The present work aims to clarify this overlooked issue, understanding the differences between these collagens and their effect on their biological performance. 

Understanding collagen content, distribution, and type is crucial for the efficient use of a potential collagen source as well as to establish an adequate collagen isolation procedure. *C. reniformis*’s general morphological organization has previously been reported. However, no histological study focused on the sponge collagen content and distribution [18]. The examination of *C. reniformis* anatomy and overall microarchitecture allowed us to determine that the ectosome, although representing a smaller portion of the sponge’s whole body, was richer in collagen than the choanosome (Figure 1), which is in agreement with previous observations [25,26,44]. Masson’s Trichrome and Picrosirius red staining revealed that along with ectosome, the zone surrounding the canals of the aquiferous system present throughout the whole sponge body was an area of heavy collagen deposition (Figure 1C–F). These results are in accordance with previous observations, which demonstrate that collagen surrounding the water canals is required to support their intricate network [18,45]. Additionally, Picrosirius red staining revealed that collagen fibers were bright yellow and thicker in the ectosome and around the water canals, while in the choanosome, they were greenish and thinner (Figure 1E,F), and it has been described that type I collagen fibrils aggregate forming thick bundles while type IV collagen forms a web rather than fibrils, thus being thinner [46]. Furthermore, the dot blot results indicated that the mesohyl of ectosome possessed collagen type I and IV while the mesohyl of choanosome was mainly constituted by collagen type IV (Figure 3B). Taking all these data into account, it is clear that distinct *C. reniformis* body regions possess different collagen types; the ectosome is constituted by collagen types I and IV, while the choanosome is almost completely comprised of collagen type IV. We hypothesize that the residual amount of collagen type I detected in the choanosome is most likely the collagen surrounding and supporting the elements of the aquiferous system since the collagen fibers in those areas are thick, similar to those encountered in the ectosome. This had never been reported earlier and is valuable information for the development of future TERM applications since different tissues are constituted by distinct collagen types, and the biomaterials developed for regenerative purposes are more promising when matching the target tissue composition [5].

A comprehensive characterization of the isolated collagens is fundamental to comprehending their properties and purity, especially when aiming to employ them in biomaterials for TERM strategies. To be used in a biomaterial, collagen must undergo a strict characterization process to ensure it matches the requirements of the desired biomedical application. FTIR was performed to confirm the chemical composition of the isolated collagens, and both samples presented a similar spectrum (Figure 2A). In fact, both FTIR spectra are in accordance with previously published *C. reniformis* collagen spectra and are very similar to other previously described marine collagens obtained from other sources, indicating an apparent chemical composition conservation [23,24,26,47,48,49,50]. Curiously, *C. reniformis* collagen has been reported to have similar spectra to vertebrate collagen and, more specifically, to fibrillar calf skin type I collagen [24,26]. In the present work, collagen type I was detected by dot blot in ectosome and residually in choanosome, thus validating this observation.

The conservation of the triple helix conformation on both isolated collagens was confirmed via CD, assuring that the collagen isolation procedure employed did not denature the protein (Figure 2B). *C. reniformis* collagen CD spectra obtained from samples isolated using water acidified with carbon dioxide did not present positive peaks, indicating the presence of random coils, which is known to diminish the performance of biological molecules [20,28,37,38]. In this sense, the *C. reniformis* collagen isolation protocol utilized in the present work is clearly advantageous as it preserves collagen triple helix conformation. Additionally, the CD spectra obtained in this study were in agreement with collagen spectra from other marine sponges, cnidarians, and vertebrates [51,52,53]. The amino acid composition of the isolated collagens was mostly similar, the most significant exceptions were OHpro content and the hydroxylation degree, which were higher in the ectosome collagen (Figure 2C). Pyrrolidine amino acids (Pro and OHpro) are associated with the collagen triple helix conformation thermal stability; thus, this result indicates that, theoretically, ectosome collagen had a higher thermal stability than choanosome collagen. However, this hypothesis must be confirmed using more specific techniques, such as thermogravimetric analysis or differential scanning calorimetry. Nonetheless, when considering employing collagen for biomedical applications it is quite beneficial to have higher thermal stability as this protein is structurally fundamental to support various connective tissues and is intended to be employed at body temperature [5,9]. In this sense, ectosome collagen could be preferred over choanosome collagen. Additionally, Gly is expected to be the most abundant amino acid, accounting for roughly one-third of the amino acid residues in mammal-derived collagen triple helix, as this is characterized by sequence repetitions of the triplets Gly-X-Y, where X and Y are often proline (Pro) and hydroxyproline (OHpro), respectively [54]. However, the values obtained for both collagens were lower, in line with previous observations [20,27,28], which can be explained by glycoproteins known to be strongly associated with collagen, the existence of non-collagenous spongin-specific proteins containing halogen groups or by major non-triple-helical sections in the analyzed collagen molecules [24,27,55]. Other previously reported amino acid profiles of *C. reniformis* collagen are in agreement with the obtained results, presenting remarkable similarities in nearly all amino acid contents, as the general composition is maintained [26,56]. The only exception regarding *C. reniformis* collagen amino acid composition was found in the work of Heinemann and co-workers, which presented much lower values of Gly and OHpro, probably due to the existence of major non-triple-helical sections in the collagen molecules as a result of the collagen isolation process employed [24]. The SDS-PAGE profiles of ectosome and choanosome collagens were similar, as both samples were only detected on the stacking gel (Figure 3A). Even though the separation gel was prepared with a low acrylamide composition (7.5%) to facilitate the sample’s penetration, due to their heavy molecular weight, they were not able to migrate to the separation gel, indicating that both collagens preserved their fibrillar structure even after denaturation procedure have been applied. Similar results have been previously presented, in which *C. reniformis* collagen large fibrils remained trapped in the stacking gel [20,23]. However, other *C. reniformis* collagen SDS-PAGE profiles reported in the literature contrast with these results, presenting bands around 110 and 100 kDa [23,30]. These differences are attributed to different collagen isolation methods which do not preserve collagen in its fibrillar conformation, since Pozzolini and colleagues treated isolated collagen fibrils with trypsin to obtain collagen hydrolysates [30], while Fassini and co-workers, although using a similar protocol to the one employed in this work, significantly increased the *C. reniformis* incubation time in disaggregation solution [23]. Additionally, as previously described, Coomassie blue was not able to stain the collagen samples; staining was only achieved with a glycoprotein staining kit, indicating the highly glycosylated nature of the collagen [23]. High glycosylation levels are known to demonstrate interesting bioactivities such as antioxidant and antimicrobial, further suggesting the benefits of *C. reniformis* collagen for TERM applications [57].

Despite it being reported that *C. reniformis* possesses collagen similar to type I and IV, no specific antibody detection has been performed so far. In the present work, it was demonstrated by dot blot that ectosome collagen was composed of collagen type I and IV, while choanosome collagen was mostly composed of collagen type IV, having only residual amounts of collagen type I, whereas type II collagen was not detected in either body part (Figure 3B). The FTIR spectra similarity between *C. reniformis* collagen extracted from the whole body and bovine type I collagen proposed by Heinemann et al. was presently validated, although this collagen type was detected predominantly in the ectosome [24]. The previously reported high structural resemblance of the non-fibrillar collagen encoded in *C. reniformis* to type IV basement membrane collagen was also supported by the dot blot results [25]. The previously reported findings state that non-fibrillar collagen presented a higher expression in ectosomes than in choanosomes, seeming to be involved in the formation of the *C. reniformis* ectosome [25]. In fact, genomic studies have detected collagen type IV sequences in a homoscleromorph sponge [58]. Accordingly, in the obtained dot blot results, collagen type IV appeared to be present in a higher quantity in ectosome than in choanosome collagen, with a slight concentration difference among both sponge body parts. The collagen isolation yield was similar in both sponge body parts, although it could be expected that the yield from ectosome would be superior since it has been characterized as possessing higher amounts of both fibrillar and non-fibrillar collagen than choanosome [25,26,44]. This could be due to the distinct accessibility of collagen in the different sponge body parts, as the ectosome presents a densely packed collagen matrix that possibly does not allow effective collagen isolation, while in the choanosome, the collagen matrix is looser and easily accessible. Nonetheless, the obtained yield of around 20% was identical to other *C. reniformis* collagen isolation results reported in the literature [29]. Using a similar collagen isolation procedure, Gokalp and colleagues obtained yield values ranging from 14.5% to 35.5% when isolating collagen from *C. reniformis* aquacultured under different conditions [59], while water and carbon dioxide collagen extraction procedures using this marine sponge, although faster, reported an isolation yield merely around 10% [20,28]. Additionally, Pozzolini and co-workers compared different *C. reniformis* collagen isolation methods and obtained yields ranging from 0.02% to 35% [22]. Considering that it has been determined that 30% of the *C. reniformis* freeze-dried mass is collagen, the collagen yield values reported in the current work are promising [27]. Although not being the highest yield reported in the literature, the fact that intact and highly glycosylated collagen fibers were obtained employing this collagen isolation protocol is extremely valuable. The possibility of obtaining intact collagen fibers from this marine sponge, together with its high collagen content and sustainable mariculture, makes it an attractive collagen source. These results conclusively elucidate the location and prevalence of the collagen types present in *C. reniformis*, an important step toward the valorization of this important marine collagen source, supporting the future development of tissue-specific biomedical applications.

The biological performance evaluation of the isolated collagens is essential to determine their applicability for the development of TERM strategies. In that sense, the cytotoxicity of both isolated collagens was determined (Figure 4 and Figure 5). All ectosome collagen concentrations tested were suitable for fibroblast culture, presenting an increase in cell metabolic activity and live cells over time. In fact, higher concentrations of this collagen had a better biological performance than the control. In these cases, it was determined that it was a suitable and highly promising biomaterial for the development of TERM applications due to its beneficial action on cell metabolism and proliferation. However, using choanosome collagen, the lower cell metabolic activity and number of live cells detected when compared with the control was not encouraging. This adverse effect was observed mostly at 72 h and aggravated with the highest collagen concentration tested, as with 2 mg/mL, the inhibitory effect on the number of live cells and on cell metabolism was pronounced. Since this negative impact on cell metabolic activity was correlated with collagen concentration it may be associated with the presence of cytotoxic compounds in the extract. In fact, marine sponges are widely recognized as a source of cytotoxic compounds with potential antitumoral interest [60]. It has been previously described as the identification of a novel cytotoxic protein present in *C. reniformis*, designated chondrosin, which has selective activity against specific tumor cell lines, including L929 [61]. The extract from which this protein was isolated was obtained from the whole sponge, where choanosome mass is predominant, which may explain the fact that the cytotoxic effect was only detected in the choanosome collagen. Nonetheless, the presence of harmful compounds should not be considerable since the cytotoxic effect was more severely detected in the highest choanosome concentration tested. However, it can be assumed that the cytotoxic effect will be intensified at higher concentrations. Collagen isolated from the whole *C. reniformis* body using carbon dioxide and acidified water did not present any cytotoxic effect, suggesting that this isolation technique probably denatures chondrosin and other possible cytotoxic compounds, as it also destroys the triple helix conformation of the isolated collagen [20]. Marine collagen hydrolysates obtained from *C. reniformis* collagen extracts purified by HPLC have been tested in vitro for wound-healing application with fibroblasts and keratinocytes and showed no degree of toxicity, stimulating cell growth [30]. Additionally, *C. reniformis* collagen-based membranes have been developed and considered suitable for TERM purposes, as they showed compatibility with both fibroblast and keratinocyte cell cultures [22,29]. These membranes were produced using collagen isolated from the whole sponge body; however, it was necessary to partially remove some of the polysaccharidic components that were co-extracted with sponge collagen to improve the biocompatibility of the structures [22,29]. These observations are in agreement with our findings since the described carbon dioxide and acidified water collagen isolation is a harsh process likely to destroy some compounds during the procedure, the collagen hydrolysates were HPLC-purified, thus having no other possibly cytotoxic compounds present, and the collagen used to produce the membranes, although isolated from the whole sponge body, which is mainly constituted by choanosome mass, required an extra purification step to improve biocompatibility.

Altogether, our data demonstrate that there are significant differences between collagen isolated from different body regions of *C. reniformis*. The most relevant dissimilarities were ectosome-derived collagen being constituted by collagen type I and IV and presenting cytocompatibility on fibroblast culture, while choanosome-derived collagen was mainly composed of collagen type IV and presented marked cytotoxic effects at 2 mg/mL concentration. The identification of different collagen types and their location is critical for the development of specific TERM applications aimed at different tissues. For instance, the presence of collagen type I may be beneficial for bone regeneration strategies, while collagen type IV may be advantageous for skin regeneration applications.

## 4. Materials and Methods

### 4.1. Reagents

All reagents were purchased from Sigma-Aldrich (St. Louis, MO, USA) unless otherwise stated.

### 4.2. Sample Collection and Storage

*C. reniformis* were collected by snorkeling near the Marine Station Endoume, Marseille, France, from a depth of 4 m, fixed in 4% formaldehyde in seawater, and transported to the facilities of the University of Minho, Portugal, for further histological analysis. *C. reniformis* collected by snorkeling at Pina Reef in Kas-Kekova Special Environmental Protected Area, Turkey, from a depth of 5 and 20 m, were provided by partners of Wageningen University & Research, Wageningen, The Netherlands, frozen and transported in dry ice containers to the facilities of University of Minho, Portugal, where they were stored at −20 °C until further use for collagen isolation.

### 4.3. C. reniformis Histological Characterization

Specimens were fixed overnight by 4% formaldehyde in seawater at 4 °C. Histological sections specimens comprising the sponge’s whole body, thus including the ectosome (outer cortex) and choanosome (inner part), were embedded in paraffin wax and cut on a Leica RM2255 Fully Automated Rotary Microtome (Leica, Wetzlar, Germany) at 10 μm. Cross-transversal paraffin sections were stained with Hematoxylin-eosin (H&E) for tissues’ general overview and with Masson’s trichome and Picrosirius Red (Abcam, Cambridge, UK) for specific collagen observation [62,63]. Masson’s trichome stains collagen blue and cytoplasm red; Picrosirius Red stains collagen green, red, or yellow under polarized light, depending on fiber thickness and packing [62,64]. H&E and Masson’s trichome-stained semi-thin sections were observed under a Leica DM750 microscope (Leica, Wetzlar, Germany), and Picrosirius red stained semi-thin sections were observed with an Axio Observer (Carl Zeiss, Jena, Germany) under polarized light. 

### 4.4. Collagen Isolation

Ectosome and choanosome collagen isolation were performed separately, using a methodology previously described [59]. Briefly, marine sponge samples were thawed, exogenous materials were removed via rinsing with dH_2_O, and the ectosome was separated from the choanosome, cut into small pieces, and left under stirring in a disaggregating solution (50 mM Tris–HCl buffer pH 7.4, 1 M NaCl, 50 mM EDTA, and 100 mM 2-mercaptoethanol) for 5 days. The collagen solution (CS) was filtered and extensively dialyzed for 7 days with 2 dialyzing buffer changes per day (CS/dialyzing buffer ratio 1:1000) against dH_2_O. The suspension was first centrifuged for 10 min at 1200× *g* to further remove cell debris, sand particles, and other exogenous materials and then centrifuged for 30 min at 12,100× *g* to collect the collagen from the suspension, yielding pellets containing collagen. All steps for collagen extraction were carried out at 4 °C. Isolated collagen was freeze-dried and stored at room temperature until further use.

### 4.5. C. reniformis Collagen Characterization

#### 4.5.1. Fourier Transform Infrared in Attenuated Total Reflection Mode (FTIR-ATR)

Infrared spectra of collagens were obtained via Fourier transform infrared spectroscopy (FTIR) under attenuated total reflectance (ATR) using freeze-dried collagen. FTIR-ATR measurements were performed employing an IR-Prestige-21 spectrophotometer (Shimadzu Scientific Instruments, Columbia, MD, USA) equipped with a diamond crystal. Each infrared spectrum was an average of 32 scans collected at 2 cm^−1^ resolution in the wavenumber region of 4000–500 cm^−1^ at room temperature.

#### 4.5.2. Circular Dichroism

To determine the protein conformation of the isolated collagen, circular dichroism (CD) analysis was performed (J1500 CD spectrometer, Jasco, Tokyo, Japan) using a quartz cylindrical cuvette with a path length of 2 mm (Hellma Analytics, Hellma, Germany). For each measurement, the cuvette was filled with 600 μL of 0.1 mg/mL collagen dissolved in dH_2_O. CD spectra were obtained by continuous wavelength scans from 180 to 240 nm (average of three scans) at a scan rate of 50 nm/min.

#### 4.5.3. Amino Acid Analysis

The amino acid content of collagen was determined via quantitative analysis using a Biochrome 30 amino acid analyzer (Biochrome Ltd., Cambridge, UK). Briefly, the collagen was completely hydrolyzed, and the resultant amino acids were separated by an Ion Exchange column. After derivatization by ninhydrin, the obtained samples were analyzed at two wavelengths: 440 nm and 570 nm. To determine the concentration of amino acids in the sample, a norleucine standard was used. The percentage of collagen hydroxylation was calculated according to the following equation, in which pyrrolidine amino acid content was the sum of hydroxyproline (OHpro) and proline (Pro) amino acids:

$$Hydroxylation\left(\%\right)=\frac{OHpro\;content}{pyrrolidine\;amino\;acid\;content}\times 100$$

#### 4.5.4. Sodium Dodecyl Sulfate-Polyacrylamide Gel Electrophoresis (SDS-PAGE)

To evaluate protein molecular weight and purity, SDS-PAGE was performed using reagents from the SDS-PAGE Gel Preparation Kit and cast on a Bio-Rad Mini Protean II System (Bio-Rad, Hercules, CA, USA). Freeze-dried collagen solubilized in deionized water (dH_2_O) at 1 mg/mL was mixed with loading buffer (1:1 *v*/*v*) and heated for 2 min at 100 °C to denature the proteins. The SDS gel was composed of 7.5% separation and 4% stacking gel and was loaded with 20 μg of each collagen sample as well as 4 μL of protein marker (Page Ruler Prestained protein ladder, 10 to 250 kDa—Thermo Fisher Scientific, Waltham, MA, USA). After electrophoresis at 90 V, Glycoprotein Stain (Pierce^®^ Glycoprotein Staining Kit—Thermo Fisher Scientific, Waltham, MA, USA) was performed according to the manufacturer’s instructions to stain glycosylated proteins.

#### 4.5.5. Dot Blot

To detect the presence of collagen type I, II, and IV on the isolated collagen samples, a dot blot analysis was performed. Collagen samples were dissolved at 1 mg/mL in dH_2_O, and 1, 2, and 3 μL drops were spotted onto a nitrocellulose membrane. For a negative control, the same amounts of bovine serum albumin (BSA) were also dotted in the membrane. Non-specific sites were blocked by soaking the membranes in 5% bovine serum albumin (BSA) dissolved in Tris-buffered saline with Tween 20 (TBS-T) solution. The primary antibodies used were Anti-Collagen I antibody (ab233639, Abcam, Cambridge, UK), Anti-Collagen II (ab209865, Abcam, Cambridge, UK) and Anti-Collagen IV antibody (ab6586, Abcam, Cambridge, UK), which were conjugated with adequate secondary antibody (Anti-rabbit IgG, HRP-linked Antibody, Cell Signaling Technology, Danvers, MA, USA). Primary antibodies and secondary antibodies were diluted at a ratio of 1:1000 and 1:20,000, respectively. Chemiluminescence detection was performed using the Clarity Western ECL substrate (Bio-Rad, Hercules, CA, USA) and the Odyssey Fc Imaging System (LI-COR Biosciences, Lincoln, NE, USA).

#### 4.5.6. Isolation Yield

The isolation yield was calculated for both collagen parts (ectosome and choanosome) as the ratio of freeze-dried collagen isolated per wet weight of *C. reniformis* biomass, according to the following equation:


$$Yield\;of\;collagen\left(\%\right)=\frac{weight\;of\;collagen\;(g)}{weight\;of\;wet\;C.\;reniformis\;biomass\left(g\right)}\times 100$$

#### 4.5.7. In Vitro Cytotoxicity Assessment 

In vitro cellular assays were performed to assess the potential cytotoxicity of *C. reniformis* collagen isolated from ectosome and choanosome. Murine fibroblasts cells (L929 cell line; ATCC CCL-1) were maintained in Dulbecco’s Modified Eagle’s Medium (DMEM) low glucose supplemented with sodium bicarbonate (3.7 g/L), 10% fetal bovine serum (FBS) (Thermo Fisher Scientific, Waltham, MA, USA), 1% antibiotic–antimycotic solution (Thermo Fisher Scientific, Waltham, MA, USA) and in a humidified controlled environment (37 °C, 5% CO_2_). Before confluence, cells were trypsinized using TrypLE Express (Thermo Fisher Scientific, Waltham, MA, USA), and 1 × 10^4^ cells/well were seeded in 48-well plates. To avoid microbial contamination, powdered collagen extracts were sterilized using ultraviolet irradiation for 1 h, dissolved in the complete medium at different concentrations (0.25, 0.5, 1, and 2 mg/mL), and added to cells 24 h after seeding. A negative control (untreated cells) was incubated under the same conditions. 

The metabolic activity of cells after incubation with collagen extracts was determined by the MTS assay (CellTiter 96 Aqueous One Solution Cell Proliferation Assay, Promega, Madison, WI, USA). After 24, 48, and 72 h of incubation, the culture medium was removed, and cells were rinsed in PBS. A mixture of culture medium (without FBS and phenol red) and MTS reagent (5:1 ratio) was added to each well and left to incubate for 3 h in a humidified atmosphere (37 °C, 5% CO_2_). Absorbance intensity is directly proportional to the metabolic activity and was measured at 490 nm using a microplate reader (Synergy HT, Biotek, Winooski, VT, USA).

Cell viability after incubation with collagen extracts was assessed by live/dead assay. Calcein-AM (Thermo Fisher Scientific, Waltham, MA, USA) and propidium iodide (PI) staining were performed after 24, 48, and 72 h of incubation with collagen extracts. Briefly, the culture medium was removed, and calcein-AM and PI at a final concentration of 1 μg/mL and 5 μg/mL in the culture medium, respectively, were added to cells. After 10 min in the dark at 37 °C in the CO_2_ incubator, samples were immediately examined using a Zeiss Axio Imager Z1 fluorescence microscope (Carl Zeiss, Jena, Germany). Cell viability (%) was calculated by counting the live cells on the 48-well plates with reference to the counted live cells of control at 24 h generated by LAS X Image Analysis (3D) software (version 3.0.16120) and using the following equation:


$$Cell\;viability\;(\%)=\left(\frac{Live\;cells\;(green)}{Live\;cells\left(green\right)\;of\;control\;at\;24\;h}\right)\times 100$$

The quantification results were presented as mean ± SD of 3 independent experiments with 2 replicates and at least 3 images per replicate.

### 4.6. Statistical Analysis

Data were presented as the mean ± standard deviation (SD) of three independent experiments. Statistical analyses were performed using GraphPad Prism 8.0.1 software (La Jolla, CA, USA). For cytotoxicity assays, data normality was evaluated by the Shapiro–Wilk test. For the two-group comparison, a two-way ANOVA test was performed, followed by Tukey’s test. Statistical significance was defined as *p*-value less than 0.05 (*p* < 0.05). 

## 5. Conclusions

For the first time, an in-depth evaluation of collagen isolated from different body zones of the collagen-rich marine sponge *Chondrosia reniformis* was performed. The collagen isolation procedure presented a similar isolation yield, and both collagens displayed equivalent FTIR spectra and were isolated in their fibrillar form while preserving their triple helix conformation. However, ectosome collagen had a higher hydroxylation degree than choanosome collagen, which may confer greater thermal stability and was composed of collagen type I and IV, while choanosome collagen was composed mostly of collagen type IV. Nevertheless, the most striking distinction between both isolated collagens was regarding their biological performance. Ectosome collagen enhanced cell metabolic activity and proliferation, an effect more evident at higher concentrations, whereas choanosome collagen had the opposite impact. There was an inhibitory effect detected at all tested concentrations, but at 2 mg/mL, the cytotoxic effect on cell metabolism and proliferation was severe. These data indicate that ectosome collagen was the most suitable for the production of TERM applications due to its biocompatibility, while choanosome collagen may be advantageous for designing novel cancer therapies due to its harmful effect on tumor cell lines. This information is essential to support and encourage the development of future biomedical applications using this sustainable collagen source. Future studies should include the development of TERM applications using ectosome-derived collagen, particularly scaffolds for the tridimensional culture of cells, thus assessing its suitability for human tissue engineering, enabling the valorization of marine resources in the context of blue biotechnology.

## Figures and Tables

**Table 1:** Collagen isolation yields.

*C. reniformis* Collagen	Isolation Yield (%)
Ectosome	20.0
Choanosome	20.2

## Data Availability

The data presented in this study are available on request from the corresponding author due to privacy reasons.
